# Contribution and performance of female Community-Directed Distributors in the treatment of onchocerciasis with Ivermectin in Sub-Saharan Africa: a systematic review

**DOI:** 10.11604/pamj.2015.20.188.3337

**Published:** 2015-02-27

**Authors:** Marius Zambou Vouking, Violette Claire Tamo, Carine Nouboudem Tadenfok

**Affiliations:** 1 Center for the Development of Best Practices in Health, Yaounde Central Hospital, Henri-Dunant Avenue, Messa, Yaounde, Cameroon; 2 Central Regional Delegation, Ministry of Public Health, Yaoundé, Cameroun; 3 School of Health Sciences, Catholic University for Central Africa, Yaounde, Cameroon

**Keywords:** Systematic review, contribution, women, Community Distributors, onchocerciasis, Sub-Saharan Africa

## Abstract

The African Program for Onchocerciasis Control (APOC) was launched in 1995 with the main goal being to boost the fight against onchocerciasis in Africa. In 2011, over 80 million people benefited from this intervention thanks to the contribution of 268.718 Community-Directed Distributors (CDD). These significant results obscure the role of women CDD in this fight. Indeed, the insufficient involvement of female CDD has been identified as a concern by the APOC partners early in the program. The present study aims to assess the contribution and performance of women involved in a strategy to control onchocerciasis by community-directed treatment with ivermectin in sub Saharan Africa. We searched the following electronic databases from January 1995 to July 2013: Medline, Embase (Excerpta Medica Database), CINAHL (Cumulative Index to Nursing and Allied Health Literature), LILAS (Latin American and Caribbean Literature on Health Sciences), International Bibliography of Social Sciences, Social Services Abstracts, and Sociological Abstracts. Two research team members independently conducted data extraction from the final sample of articles by using a pre-established data extraction sheet. The primary outcome was the contribution of female CDD in the control of onchocerciasis by community-directed treatment with Ivermectin. Of 25 hits, 7 papers met the inclusion criteria. For the management of onchocerciasis, female CDDs are elected by the health committee from the communities they will serve. The significant proportion of those treated (about 61%) were women, although only 24% of CDDs were women. Many community members reported that women were more committed, persuasive and more patient than men in the distribution of ivermectin. Some studies have identified underutilization of female CDD as one reason for the limited effectiveness or, in some cases, pure failure related to the distribution of Ivermectin interventions in the fight against onchocerciasis in sub-Saharan Africa. Evidence from this review suggests that female CDD contribute to the treatment of onchocerciasis with Ivermectine in sub-Saharan Africa. Large-scale rigorous studies including Randomized controlled trials (RCTs) are needed to compare Community-Directed intervention involving men and women CDDs.

## Introduction

Onchocerciasis is a neglected tropical disease caused by the parasite *Onchocerca volvulus*, which is transmitted by the black fly [1]. The *Simulium* fly that transmits onchocerciasis breeds in fast-flowing rivers, giving rise to the disease's common name "river blindness" [1] (WHO, 2011). Ivermectin is a safe and effective oral microfilaricide developed since 1987 to control onchocerciasis [1]. The African Program for Onchocerciasis Control (APOC) was launched in 1995 to boost the fight against onchocerciasis in Africa initially carried out by the Onchocerciasis Control Programme (OCP) in West Africa [2]. The strategic objective of APOC is to permanently protect the remaining 120 million people at risk of this debilitating and disfiguring disease in 19 countries in Africa through the establishment of Community-Directed Treatment with Ivermectin (CDTI) that is capable of being sustained by the communities after APOC financing has ended [2]. The long-term support of onchocerciasis control together with the sustained political commitment of national governments, bilateral donors, and Non-Governmental Development Organisations (NGDOs), is a major yet unheralded public health and development success story in Africa [3].

In 2011, over 80 million people of 103 million have benefited from this intervention in 16 countries thanks to the contribution of 682.091 Community-Directed Distributors [4]. These significant results obscure the role of female Community-Directed Distributor (CDD) in this fight. Indeed, the insufficient involvement of female CDD has been identified as a concern by the APOC partners early in the program [5–8]. A CDD is a person selected by the community based on personal characteristics such as integrity, honesty and literacy, who is responsible for the procurement of the drug (Ivermectin) from the closest health center, taking census, administering medication, keeping inventory of Ivermectin, treating minor adverse medication reactions and referring more serious ones to health facilities, keeping records and delivering other health interventions [9]. Mooney [10] has reviewed the various social theories underlying different stances on equity and shown how they lead to sometimes profoundly different ways of conceptualizing it. The systematic review of Allotey and Gyapong [11] showed the important role that women can play in the fight against tropical diseases in general.

Although the achievement of gender balance may have inherent advantages, the impact of accountability on performance and opportunities for female CDD is a question not yet studied. In this context, a comparative analysis between both sexes could illuminate the contribution of gender balance in this strategy. In addition, there are opportunities to assess the effects of gender on interest and compliance with treatment for a single intervention. Data on these issues could provide evidence for the acceleration of the elimination of onchocerciasis in sub-Saharan Africa. The general objective was to assess the contribution and performance of women involved in a strategy to control onchocerciasis by Community-Directed Treatment with Ivermectin in Africa. Specific objectives were assess and compare the performance of female and male CDD regarding; coverage and speed of distribution, referral and follow-up of absentees and non eligible people; evaluate perceived benefits to communities as a result of active female involvement in community-directed intervention; and identify factors in the existing health services which promote, support or hamper female involvement.

## Methods

### Search strategy

We searched the following electronic databases from January 1995 to July 2013: Medline, Embase (Excerpta Medica Database), CINAHL (Cumulative Index to Nursing and Allied Health Literature), LILAS (Latin American and Caribbean Literature on Health Sciences), International Bibliography of Social Sciences, Social Services Abstracts, and Sociological Abstracts. The following search strategy was modified for the various databases and search engines: («contribution» OR «role» OR «impact» AND «female Community-Directed Distributors» OR «female Community Distributors» OR «female Community Health Worker» OR «Lay Health Worker» OR « female Community Health Aide » OR « female Community Worker » OR «female Village Health Worker» OR « female Barefoot Doctor» AND «treatment» OR «distribution» AND «Ivermectin» OR «Mectizan» AND «Onchocerciasis» OR «*Onchocerca volvulus*» AND «Sub Saharan Africa» OR «Endemic country»). Along with MeSH terms and relevant keywords, we used the Cochrane Highly Sensitive Search Strategy for identifying reports of articles in Pubmed. There were no restrictions to language or publication status. Our search was limited to the last nineteen years, as they correspond to the period of enactment of the APOC [2]. Prior to the APOC, efforts to control Onchocerciasis, especially with CDDs were almost inexistent.

### Study design

All study designs were eligible for inclusion provided they were on female CDDs working on onchocerciasis in sub-Saharan Africa.

### Types of studies

Randomized controlled trials (RCT), controlled before and after, uncontrolled before and after, interrupted time series, cross-sectional studies, cohorts, and case control studies.

### Types of intervention

We included female CDD delivering curative care, with or without preventive services to people for at least one year or one onchocerciasis intervention.

### Types of outcome measures

The primary outcomes were contributions of female CDD in the control of onchocerciasis by community-directed treatment with Ivermectin in Africa. Secondary outcomes included: women's knowledge on community-directed treatment with Ivermectin; the proportion of women in the community-directed treatment and the proportion of men who approve female CDDs.

### Effectiveness outcomes

Studies were included if they provided data on role and performance of female CDDs of community based intervention program on onchocerciasis for one year (or more).

### Data extraction and management

Two research team members (VCT and CNT) independently conducted data extraction from the final sample of articles by using a pre-established data extraction form. Disagreements were resolved by consensus or by arbitration of a third review author (MZV). Studies were reviewed for relevance based on the type of participants (female CDDs), interventions (distribution Ivermectin or onchocerciasis treatment) and outcome measures. We retrieved full text copies of the articles identified as potentially relevant by either one or both review authors. The flow of study selection is described in a Preferred Reporting Items for Systematic Reviews and Meta-Analyses (PRISMA) diagram [12]. Data are reported in a narrative manner.

### Assessment of quality in included studies

The included studies were not scored for quality.

## Current status of knowledge

**Figure 1:**
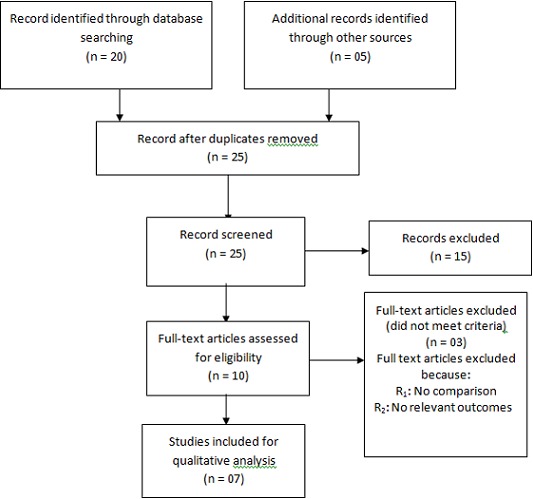
PRISMA flow diagram

Our research resulted in achieving 25 studies of which 07 were conducted in Tanzania, Cameroon, Nigeria and Uganda were included in this systematic review (Figure 1).

### The recruitment of female CDDs

Female CDDs were either elected by the officials of the health committee of the area [6, 7, 13, 14] to be nominated by members of the community [5, 8, 15]. Little information is available on their level of education. In Oyo State (Nigeria), the selection of community consensus CDD was made on the basis of criteria such as the popularity of these people, their level of education (high school degree in health studies), their honesty, their work ethic, their interest and previous experience [7]. In Tanzania, through Community-Directed Interventions (CDI), there was growing awareness of women to participate in the same position as there were women who were in the formal power networks (women leaders). Elsewhere, selection of CDDs considered gender because there was an equal opportunity for men and women who were selected as drug distributors in each hamlet. The selection of CDDs increased women's representatives in CDI activities [13].

### Training female CDDs

Female CDDs were trained annually in the distribution of Ivermectin in the program called "Community-Directed Interventions" (CDI). They have also been trained in the detection of side effects associated with taking Ivermectin and record keeping [5–8, 13, 15]. After selection, training on all four health interventions was initiated and supervised by district health personnel [5–8, 13, 15].

### Proportion of female CDDs in onchocerciasis programs

Four studies evaluated the proportion of female CDDs in Tanzania, Nigeria, Cameroon and Uganda [5–7, 13]. The study results showed that a significant proportion of those treated (about 61%) were women, although only 24% of CDDs were women [5–7, 13].

### Contribution of female CDDs in the control of onchocerciasis

The study by Brieger and colleagues [7] in Nigeria and Cameroon showed that 81% of people where CDDs were women received ivermectin compared to 78% in villages where women do not exercise CDD. The study in Tanzania shows that there was a growing awareness in the CDI for the participation of women in community-based distribution of Ivermectin [13]. The approach to mainstream gender in the selection of female CDDs increased representation of women and the distribution of Ivermectin in communities affected by CDI [13]. All but two of the women interviewed on how they perceived the performances of women and men as CDDs; 98 (38%) said that women were more likely to be patient and tolerant than men, 73 (28%) thought that men were more active than women but more impatient, 82 (32%) said that women were more committed than men and 5 (2%) said that men were rude. Further analysis revealed that those who said that they had attended the health education sessions were more likely to have been involved (or, at least, to claim they had been involved) in the selection of CDDs, the choice of the method of drug distribution and the decision-making on the time of distribution (P < 0.001) than those who said they had not attended the education sessions [13]. In Uganda, 70% of community members surveyed reported that women were more committed, persuasive and more patient than men in the distribution of Ivermectin. The men interviewed did not oppose to the distribution of ivermectin by women, on the contrary, several men said they would be happy and proud to see their wives accept this task if they were designated to do so [5, 8].

In Yola site (Nigeria) by cons, women were not initially selected as CDDs because in past experiences of Ivermectin distribution, CDDs had to go from one house to the other, which was considered inappropriate for women in this Muslim area [7]. Specific concerns about women and ethnic minorities were clarified. During the Focus Group Discussion, women typically gave comments such as the following, which showed a lack of their involvement in some villages: *"Females were not invited to the village meeting where the outcome of the meeting was discussed". "Our husbands put their heads together when the representatives came for the meeting, but we don't know what they discussed". "When those who attended the meeting got back to the village, they gave feedback only to the male village elders, but we females and the young villagers were not invited"* [7].

In the Masya and Karangara health District in Uganda, women who have been health educated were more involved CDI decision making and had better attitude and performance compared with men [6]. Face to face interviews and records indicate that female CDDs achieved as good coverage as their male counterparts and sometime better in about the same time [6]. Health education increased the number of female CDDs from 9 to 52 in Masya Health District, from 7 to 22 in Karangara District and 6 to 20 in Mukongo District [6]. In Tanzania, the Lushoto District key Informant said: *"This program values the contribution of women in the community. This is good because women are hard working and we always appreciate them"* In Kilosa district, a village leader at Zombo Playa had this to add *"Community-directed treatment with ivermectin selection considers gender balance whereby, from each hamlet they are supposed to have two CDDs, a man and a woman"* [13]. Boussinesq et al. [15] found that females aged 15 to 29 years were very busy with housekeeping activities, and therefore less disposed to come to distribution points in Cameroon.

The concept of women's empowerment has gained increased attention over the past two decades. The idea that the empowerment of women is an essential component of international development first held prominence at the International Conference on Population and Development (ICPD) in Cairo in 1994 and then again at the Fourth World Conference on Women, Beijing 1995 [16]. These conferences marked a shift from thinking of women as targets for fertility control policies to acknowledging women as autonomous agents with rights. Since 2007, APOC has implemented a strategy of gender mainstreaming which ensures on gender issues in onchocerciasis endemic countries, particularly in CDDs that occupy a prominent position in the control of onchocerciasis. Literature review results suggest several implications for policy and program efforts to scale up and sustain CDD programs in sub-Saharan African countries. First, selection, training and motivation of implementers have been amongst others, the important key issues to sustain the CDI approach. The community members select implementers with basic characteristics set up by them. Different communities have different criteria for selection of CDDs. Generally, cultural and political structure influences the selection and even the contribution of women on CDD programs. According to Clemmons et al. [17] community leaders can influence the inclusion of women as CDDs in health programs. In addition, the low participation of women in CDD decision making and community participation is at the origin of the low coverage of the distribution of Ivermectin in most endemic areas of Africa. When CDDs were not chosen by the community as part of a broad consensus process, problems of allegiance to various clans or lack of dedication to the community have emerged over time [18], women CDDs chosen by the community are more satisfied and willing to work under the conditions determined by the community and therefore have a higher level of retention. Secondly, a gender inequality approach to health is concerned with the role of gender relations in the production of vulnerability to ill health or disadvantage within health care systems and particularly the conditions which promote inequality between gender in relation to access and utilization of services. It is thus more centrally concerned with power relations and the ways in which health may also be a site of gender conflict. For example, the results of studies have shown that a significant proportion of those treated (about 61%) were women, while only 24% of CDDs were women. This imbalance in patients/CDDs ratio shows how much work remains to be done in the fight against this disease. The reasons for this disparity are related largely to cultural and religious barriers. Indeed, there remain areas of Tanzania where CDDs are still male-dominated, where community leaders decide themselves [13] or where it is culturally frowned and dangerous for women to take a role that leads them to go without escort in other people's houses as in northern Nigeria [7].

However, although the achievement of gender balance may be advantageous, this strategy goes against CDIs and although the APOC program to encourage communities, highlighting the importance of gender issues, it returns ultimately to the communities to identify those they consider most suitable for these roles. The 2012 report indicates that APOC is gradually increasing in the proportion of women in CDD affected by onchocerciasis, it increased from 25.9% (121 976 CDD women out of a total of 470 816 CDD) in 2009 to 26.5% (143 167 CDD women out of a total of 541 226 CDD) in 2010 and 29.1% (198 579 CDD women out of a total of 682,091 CDD) in 2011 [4]. Thirdly, increasing the involvement of women in the CDTI-related decision making processes and respecting, understanding and making use of the traditional social structures and social legal systems are vital preliminaries to the recruitment of women as CDDs. kinship enhanced CDTI had more female community distributors than classic CDTI, showing that the utilisation of the kinship system may be more suitable for women's involvement. Analysis showed that the presence of female CDDs might have some positive influence on coverage, especially in areas where it is difficult for men to administer treatment to women. For example, the study by Brieger and colleagues [7] in Nigeria and Cameroon showed that 81% of people where there were women CDDs received ivermectin compared to 78% in villages with no female CDDs. This difference of three points in favor of women's action is therefore an asset that decision makers could use to advance the goals set by APOC and thus accelerates the process of elimination of the disease. In 2011, the average therapeutic coverage was 77% and the average geographical coverage was 95% in endemic countries [4]. The 2008 [18] and 2006 [19] reports of the CDI showed that some community members considered preferable to perform certain interventions such as antimalarials drug by women while others were more often assigned to men.

The systematic review of Douthwaite and Ward showed the important role played by female community health workers in the use of contraceptive methods in Pakistan [20]. The 2012 evaluation report on the level of achievement of the Millennium Development Goals (MDGs) also shows the close link between achieving the goal number 3 which relate on MDGs 4 and 5, economic and political empowerment of women seeking objective 3 is important for the survival of women and children [21]. Finally, policymakers, practitioners, and researchers seeking to scale up and sustain CDD programs should develop CDD programs with attention to the 3 focal areas identified from the literature: (1) effective design and management of the female CDD program, (2) fit of the CDD program with the specific communities served, and (3) integration of females in CDD programs with the broader political, economic, and health system environments. Although promoting enabling factors and avoiding barriers within each of these 3 categories may seem straightforward, the literature suggests that this process is complex and that some degree of failure is common. Designing the right mix of female CDD selection criteria, task assignments and motivational strategies at the start of a program is challenging and initial designs may require revision over time. Policy makers and program managers should therefore remain attentive to these issues and be flexible to adapt to changing environments and constraints throughout the initial implementation and ongoing management of such programs.

### Limitations

These policy and practice recommendations of this study should be interpreted in the light of several limitations. First, many of the articles in our sample did not describe all stages of the scale-up process in equivalent levels of detail; for example, an article might discuss the early planning stages or final operational results of a CDD program but not the intermediate process of introducing CDD programs to new endemic communities. Secondly, our definition of CDDs may have excluded some untrained lay health workers. Thirdly, the lack of uniformity in the study designs and reports rendered it impossible to make a comparative assessment of their quality. Finally, only one study reported on the access and compared the performance of women with that of men CDD in the control of onchocerciasis by community-directed treatment with Ivermectin.

## Conclusion

Evidence from this review suggests that female CDDs contribute to the treatment of onchocerciasis with Ivermectine in sub-Saharan Africa. The results of some studies have identified underutilization of female CDDs as one reason for the limited effectiveness or, in some cases, pure failure related to the distribution of Ivermectin interventions. Large-scale rigorous studies, including RCTs, are needed to compare Community-Directed Intervention involving men and women CDDs.
